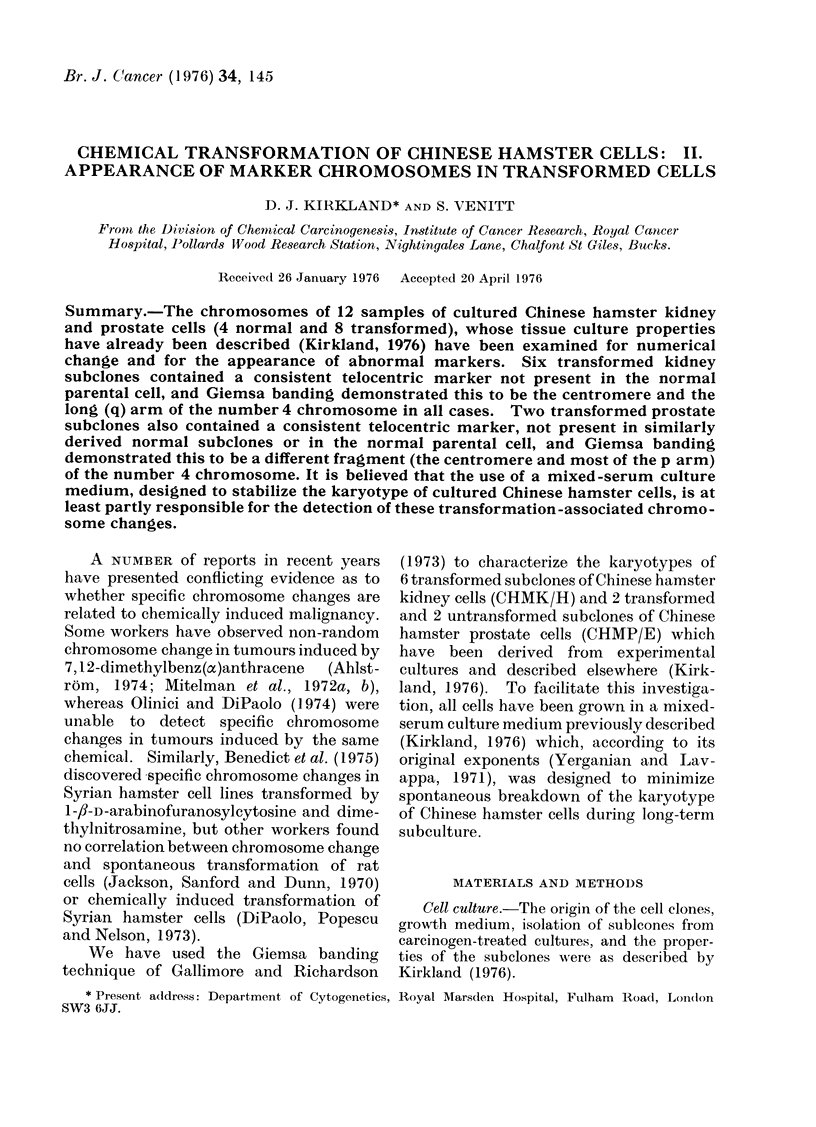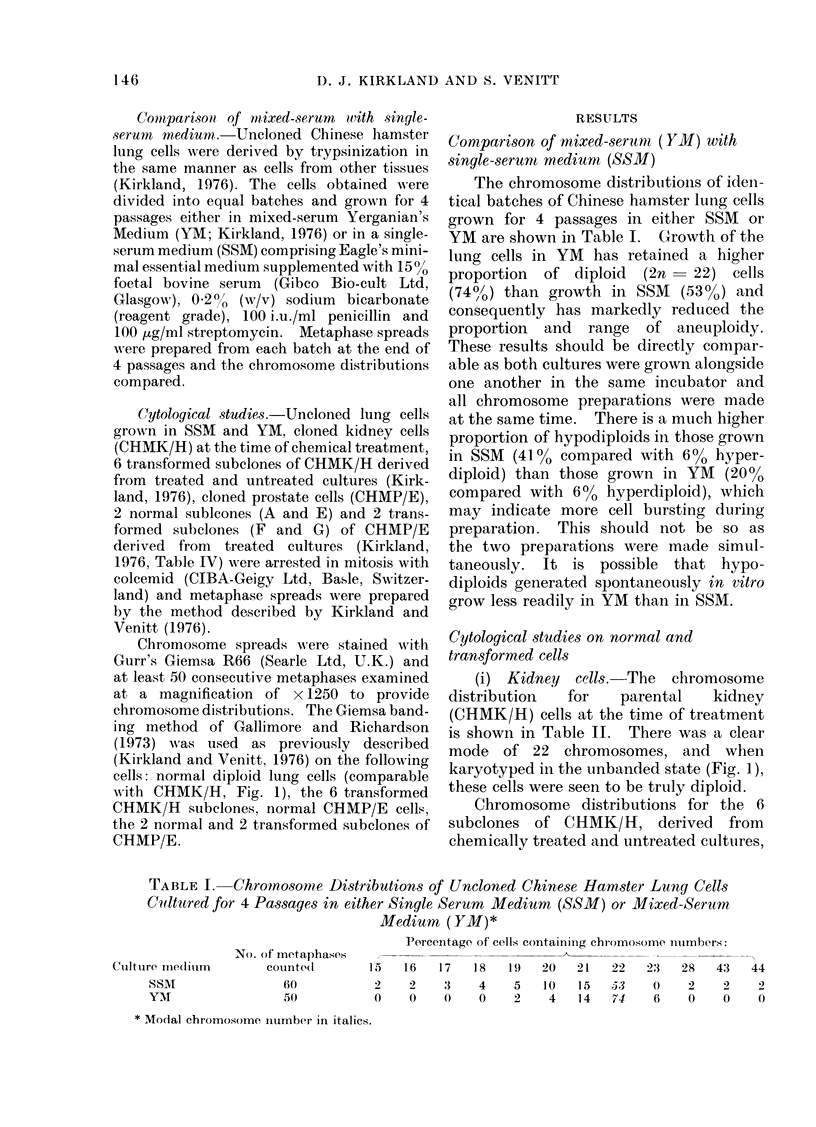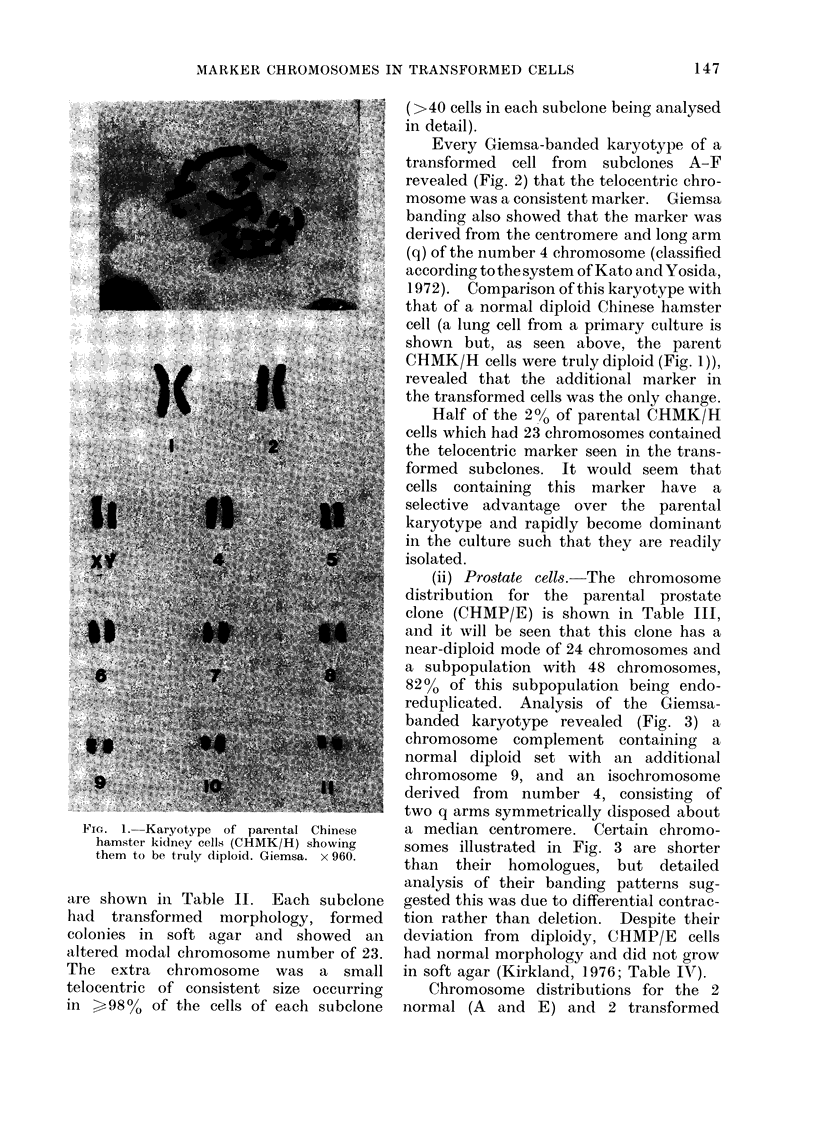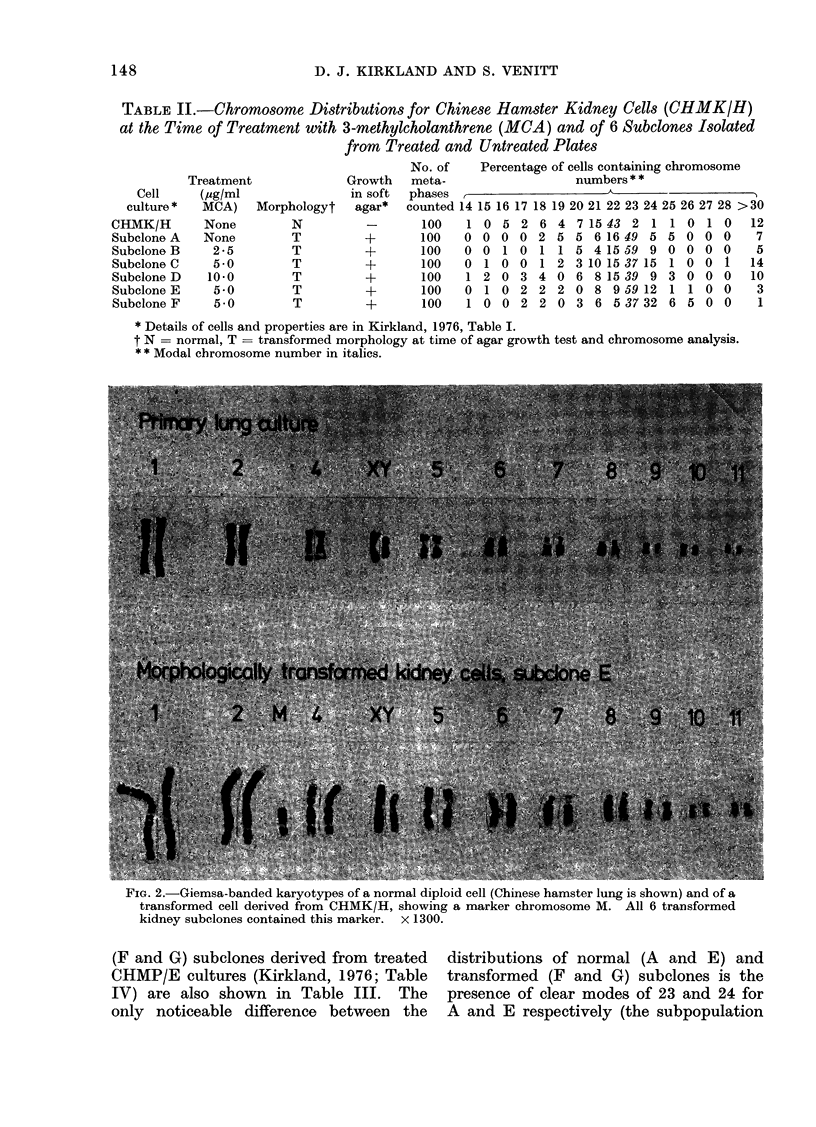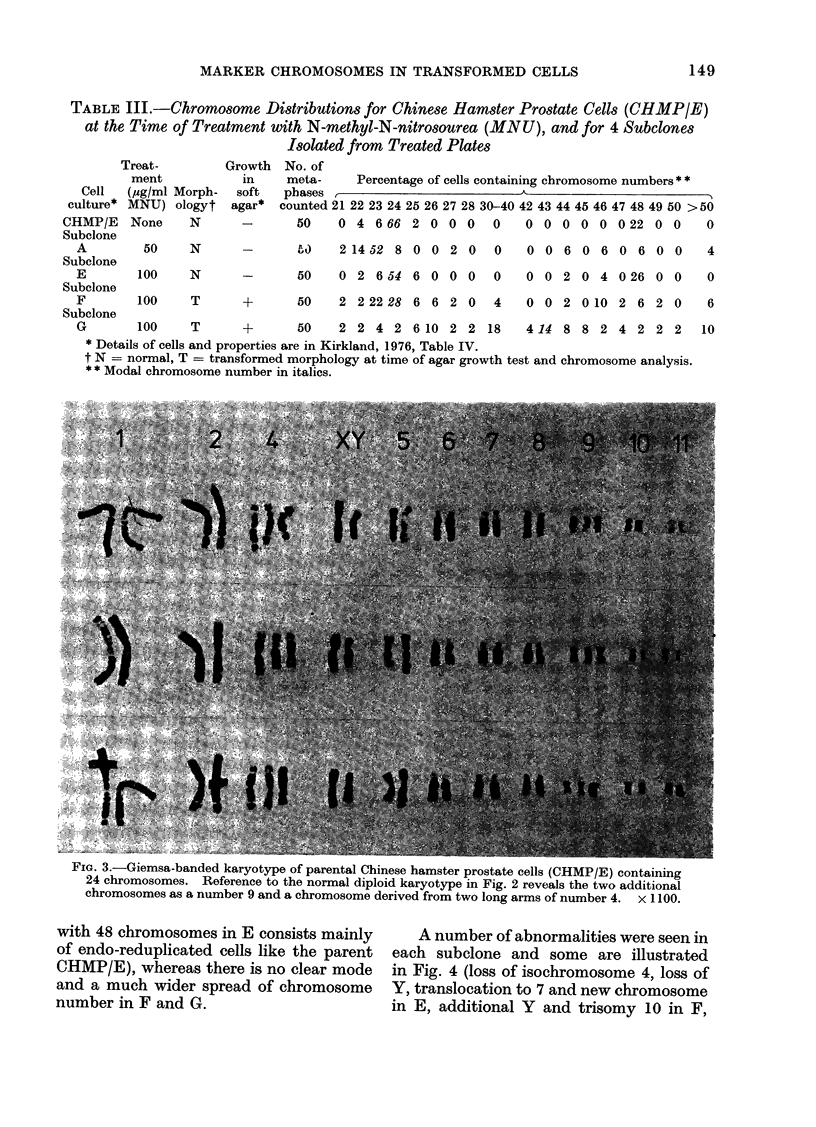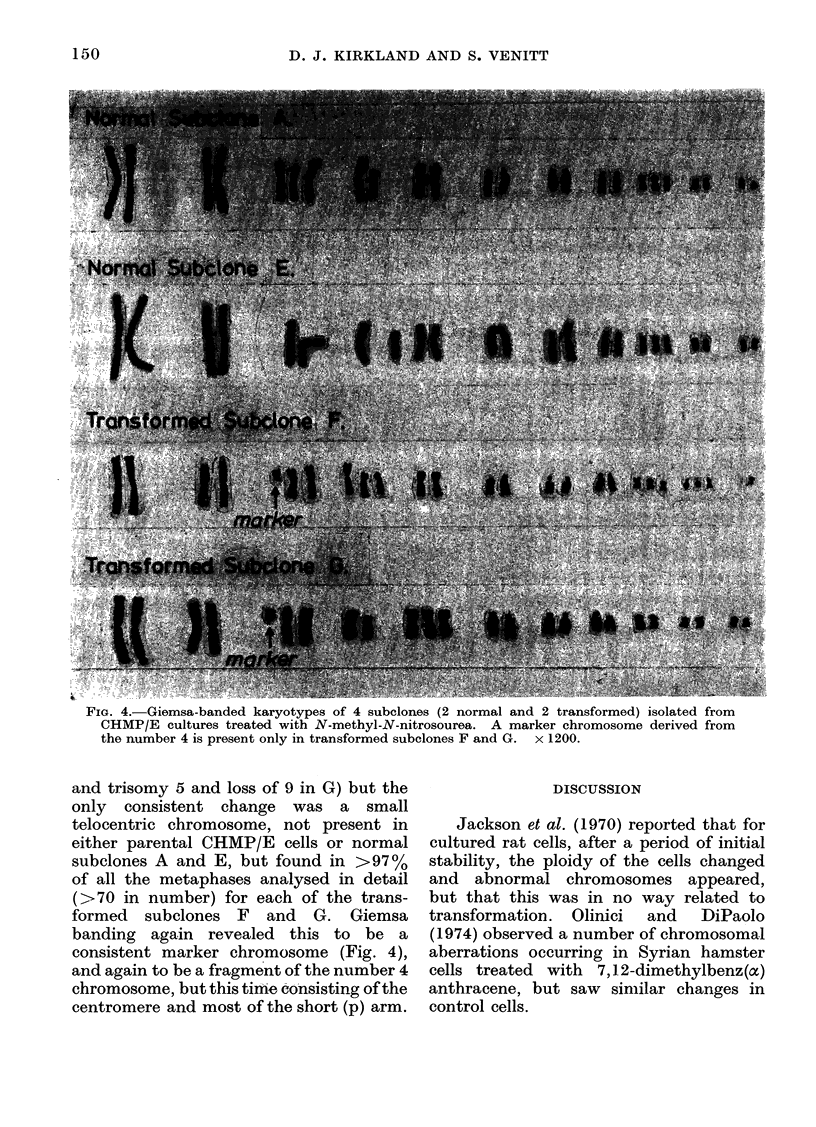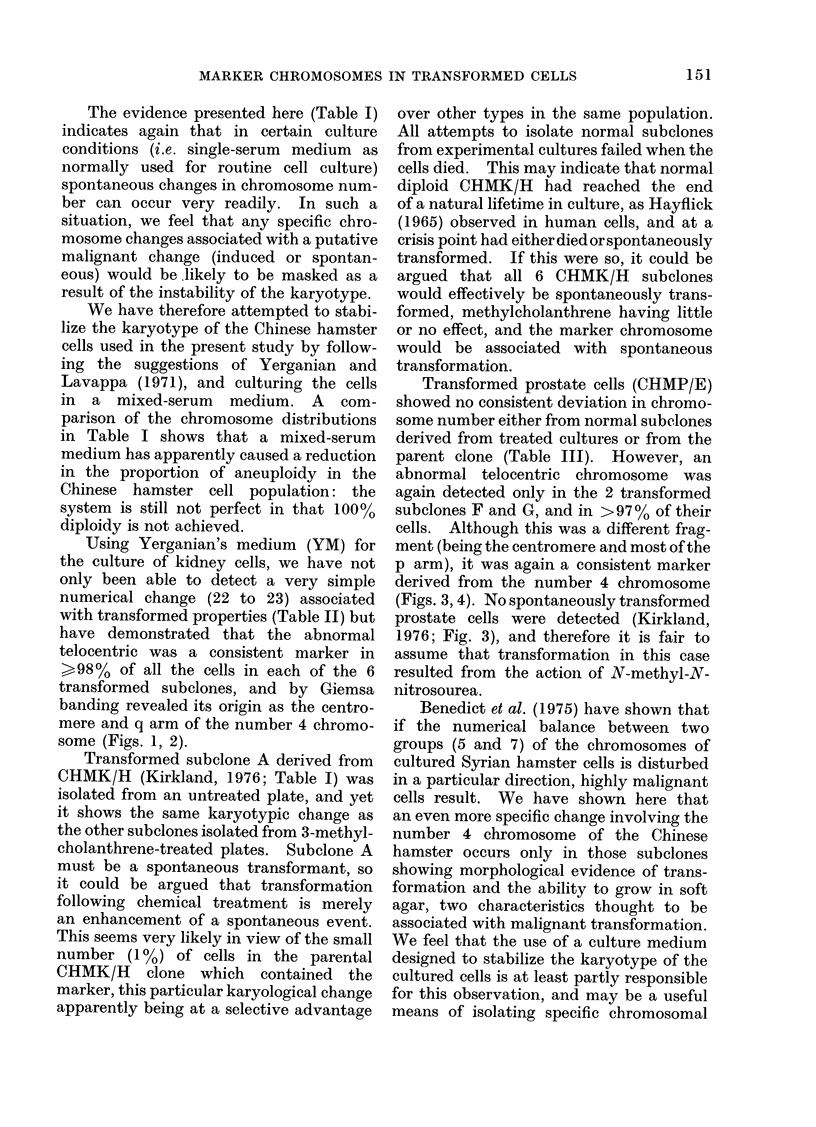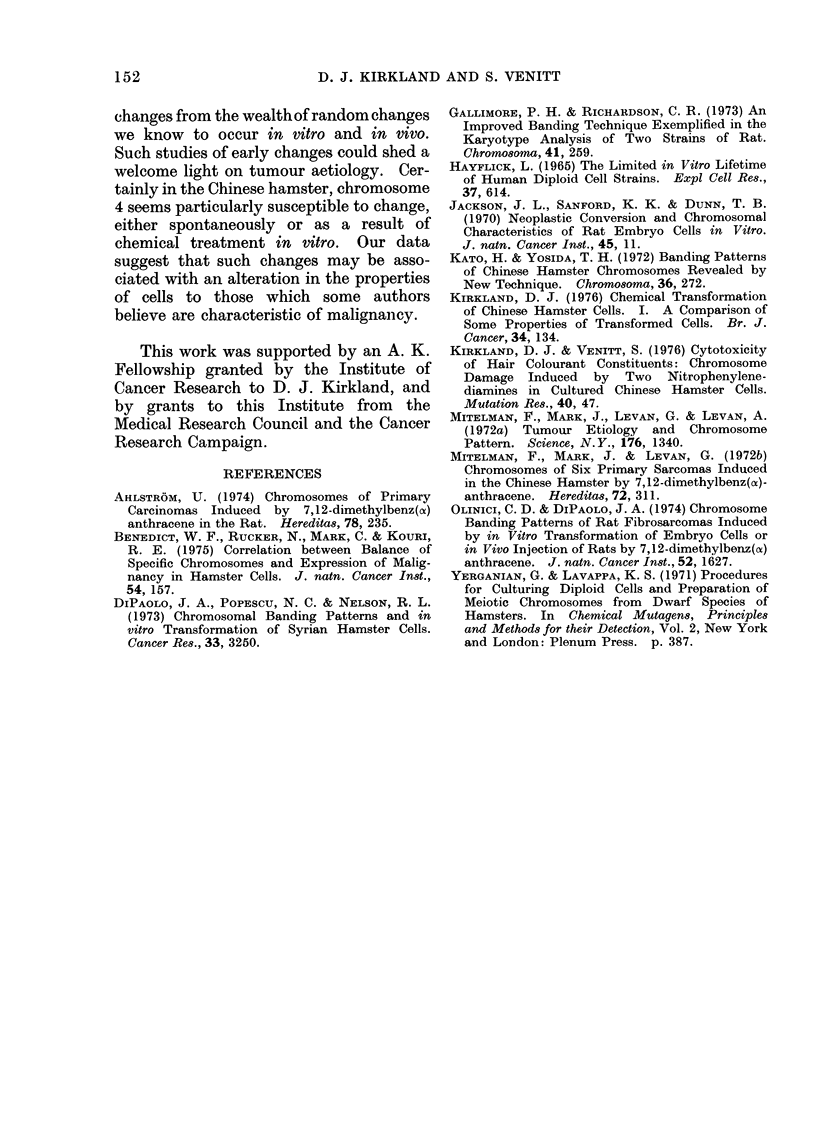# Chemical transformation of Chinese hamster cells: II. Appearance of marker chromosomes in transformed cells.

**DOI:** 10.1038/bjc.1976.136

**Published:** 1976-08

**Authors:** D. J. Kirkland, S. Venitt

## Abstract

**Images:**


*Br. J. Cancer* (1976) **34**, 145

### CHEMICAL TRANSFORMATION OF CHINESE HAMSTER CELLS: II. APPEARANCE OF MARKER CHROMOSOMES IN TRANSFORMED CELLS

D. J. KIRKLAND* AND S. VENITT

*From the Division of Chemical Carcinogenesis, Institute of Cancer Research, Royal Cancer Hospital, Pollards Wood Research Station, Nightingales Lane, Chalfont St Giles, Bucks.*

Received 26 January 1976 Accepted 20 April 1976

**Summary.—The chromosomes of 12 samples of cultured Chinese hamster kidney and prostate cells (4 normal and 8 transformed), whose tissue culture properties have already been described (Kirkland, 1976) have been examined for numerical change and for the appearance of abnormal markers. Six transformed kidney subclones contained a consistent telocentric marker not present in the normal parental cell, and Giemsa banding demonstrated this to be the centromere and the long (q) arm of the number 4 chromosome in all cases. Two transformed prostate subclones also contained a consistent telocentric marker, not present in similarly derived normal subclones or in the normal parental cell, and Giemsa banding demonstrated this to be a different fragment (the centromere and most of the p arm) of the number 4 chromosome. It is believed that the use of a mixed-serum culture medium, designed to stabilize the karyotype of cultured Chinese hamster cells, is at least partly responsible for the detection of these transformation-associated chromosome changes.**

A NUMBER of reports in recent years have presented conflicting evidence as to whether specific chromosome changes are related to chemically induced malignancy. Some workers have observed non-random chromosome change in tumours induced by 7,12-dimethylbenz(α)anthracene (Ahlström, 1974; Mitelman *et al.*, 1972*a, b*), whereas Olinici and DiPaolo (1974) were unable to detect specific chromosome changes in tumours induced by the same chemical. Similarly, Benedict *et al.* (1975) discovered specific chromosome changes in Syrian hamster cell lines transformed by 1-β-D-arabinofuranosylcytosine and dimethylnitrosamine, but other workers found no correlation between chromosome change and spontaneous transformation of rat cells (Jackson, Sanford and Dunn, 1970) or chemically induced transformation of Syrian hamster cells (DiPaolo, Popescu and Nelson, 1973).

We have used the Giemsa banding technique of Gallimore and Richardson (1973) to characterize the karyotypes of 6 transformed subclones of Chinese hamster kidney cells (CHMK/H) and 2 transformed and 2 untransformed subclones of Chinese hamster prostate cells (CHMP/E) which have been derived from experimental cultures and described elsewhere (Kirkland, 1976). To facilitate this investigation, all cells have been grown in a mixed-serum culture medium previously described (Kirkland, 1976) which, according to its original exponents (Yerganian and Lavappa, 1971), was designed to minimize spontaneous breakdown of the karyotype of Chinese hamster cells during long-term subculture.

### MATERIALS AND METHODS

*Cell culture.*—The origin of the cell clones, growth medium, isolation of sublcones from carcinogen-treated cultures, and the properties of the subclones were as described by Kirkland (1976).

* Present address: Department of Cytogenetics, Royal Marsden Hospital, Fulham Road, London SW3 6JJ.

*Comparison of mixed-serum with single-serum medium.*—Uncloned Chinese hamster lung cells were derived by trypsinization in the same manner as cells from other tissues (Kirkland, 1976). The cells obtained were divided into equal batches and grown for 4 passages either in mixed-serum Yerganian's Medium (YM; Kirkland, 1976) or in a single-serum medium (SSM) comprising Eagle's minimal essential medium supplemented with 15% foetal bovine serum (Gibco Bio-cult Ltd, Glasgow), 0·2% (w/v) sodium bicarbonate (reagent grade), 100 i.u./ml penicillin and 100 $\mu$g/ml streptomycin. Metaphase spreads were prepared from each batch at the end of 4 passages and the chromosome distributions compared.

*Cytological studies.*—Uncloned lung cells grown in SSM and YM, cloned kidney cells (CHMK/H) at the time of chemical treatment, 6 transformed subclones of CHMK/H derived from treated and untreated cultures (Kirkland, 1976), cloned prostate cells (CHMP/E), 2 normal sublcones (A and E) and 2 transformed subclones (F and G) of CHMP/E derived from treated cultures (Kirkland, 1976, Table IV) were arrested in mitosis with colcemid (CIBA-Geigy Ltd, Basle, Switzerland) and metaphase spreads were prepared by the method described by Kirkland and Venitt (1976).

Chromosome spreads were stained with Gurr's Giemsa R66 (Searle Ltd, U.K.) and at least 50 consecutive metaphases examined at a magnification of ×1250 to provide chromosome distributions. The Giemsa banding method of Gallimore and Richardson (1973) was used as previously described (Kirkland and Venitt, 1976) on the following cells: normal diploid lung cells (comparable with CHMK/H, Fig. 1), the 6 transformed CHMK/H subclones, normal CHMP/E cells, the 2 normal and 2 transformed subclones of CHMP/E.

### RESULTS

*Comparison of mixed-serum (YM) with single-serum medium (SSM)*

The chromosome distributions of identical batches of Chinese hamster lung cells grown for 4 passages in either SSM or YM are shown in Table I. Growth of the lung cells in YM has retained a higher proportion of diploid ($2n = 22$) cells (74%) than growth in SSM (53%) and consequently has markedly reduced the proportion and range of aneuploidy. These results should be directly comparable as both cultures were grown alongside one another in the same incubator and all chromosome preparations were made at the same time. There is a much higher proportion of hypodiploids in those grown in SSM (41% compared with 6% hyperdiploid) than those grown in YM (20% compared with 6% hyperdiploid), which may indicate more cell bursting during preparation. This should not be so as the two preparations were made simultaneously. It is possible that hypodiploids generated spontaneously *in vitro* grow less readily in YM than in SSM.

*Cytological studies on normal and transformed cells*

(i) *Kidney cells.*—The chromosome distribution for parental kidney (CHMK/H) cells at the time of treatment is shown in Table II. There was a clear mode of 22 chromosomes, and when karyotyped in the unbanded state (Fig. 1), these cells were seen to be truly diploid.

Chromosome distributions for the 6 subclones of CHMK/H, derived from chemically treated and untreated cultures,

TABLE I.—*Chromosome Distributions of Uncloned Chinese Hamster Lung Cells Cultured for 4 Passages in either Single Serum Medium (SSM) or Mixed-Serum Medium (YM)**

| Culture medium | No. of metaphases counted | Percentage of cells containing chromosome numbers: 15 | 16 | 17 | 18 | 19 | 20 | 21 | 22 | 23 | 28 | 43 | 44 |
|---|---|---|---|---|---|---|---|---|---|---|---|---|---|
| SSM | 60 | 2 | 2 | 3 | 4 | 5 | 10 | 15 | *53* | 0 | 2 | 2 | 2 |
| YM | 50 | 0 | 0 | 0 | 0 | 2 | 4 | 14 | *74* | 6 | 0 | 0 | 0 |

* Modal chromosome number in italics.

FIG. 1.—Karyotype of parental Chinese hamster kidney cells (CHMK/H) showing them to be truly diploid. Giemsa. ×960.

are shown in Table II. Each subclone had transformed morphology, formed colonies in soft agar and showed an altered modal chromosome number of 23. The extra chromosome was a small telocentric of consistent size occurring in $\geqslant 98\%$ of the cells of each subclone ($>40$ cells in each subclone being analysed in detail).

Every Giemsa-banded karyotype of a transformed cell from subclones A–F revealed (Fig. 2) that the telocentric chromosome was a consistent marker. Giemsa banding also showed that the marker was derived from the centromere and long arm (q) of the number 4 chromosome (classified according to the system of Kato and Yosida, 1972). Comparison of this karyotype with that of a normal diploid Chinese hamster cell (a lung cell from a primary culture is shown but, as seen above, the parent CHMK/H cells were truly diploid (Fig. 1)), revealed that the additional marker in the transformed cells was the only change.

Half of the $2\%$ of parental CHMK/H cells which had 23 chromosomes contained the telocentric marker seen in the transformed subclones. It would seem that cells containing this marker have a selective advantage over the parental karyotype and rapidly become dominant in the culture such that they are readily isolated.

(ii) *Prostate cells.*—The chromosome distribution for the parental prostate clone (CHMP/E) is shown in Table III, and it will be seen that this clone has a near-diploid mode of 24 chromosomes and a subpopulation with 48 chromosomes, $82\%$ of this subpopulation being endoreduplicated. Analysis of the Giemsa-banded karyotype revealed (Fig. 3) a chromosome complement containing a normal diploid set with an additional chromosome 9, and an isochromosome derived from number 4, consisting of two q arms symmetrically disposed about a median centromere. Certain chromosomes illustrated in Fig. 3 are shorter than their homologues, but detailed analysis of their banding patterns suggested this was due to differential contraction rather than deletion. Despite their deviation from diploidy, CHMP/E cells had normal morphology and did not grow in soft agar (Kirkland, 1976; Table IV).

Chromosome distributions for the 2 normal (A and E) and 2 transformed

TABLE II.—*Chromosome Distributions for Chinese Hamster Kidney Cells (CHMK/H) at the Time of Treatment with 3-methylcholanthrene (MCA) and of 6 Subclones Isolated from Treated and Untreated Plates*

| Cell culture* | Treatment (μg/ml MCA) | Morphology† | Growth in soft agar* | No. of metaphases counted | Percentage of cells containing chromosome numbers** 14 | 15 | 16 | 17 | 18 | 19 | 20 | 21 | 22 | 23 | 24 | 25 | 26 | 27 | 28 | >30 |
|---|---|---|---|---|---|---|---|---|---|---|---|---|---|---|---|---|---|---|---|---|
| CHMK/H | None | N | — | 100 | 1 | 0 | 5 | 2 | 6 | 4 | 7 | 15 | *43* | 2 | 1 | 1 | 0 | 1 | 0 | 12 |
| Subclone A | None | T | + | 100 | 0 | 0 | 0 | 0 | 2 | 5 | 5 | 6 | 16 | *49* | 5 | 5 | 0 | 0 | 0 | 7 |
| Subclone B | 2·5 | T | + | 100 | 0 | 0 | 1 | 0 | 1 | 1 | 5 | 4 | 15 | *59* | 9 | 0 | 0 | 0 | 0 | 5 |
| Subclone C | 5·0 | T | + | 100 | 0 | 1 | 0 | 0 | 1 | 2 | 3 | 10 | 15 | *37* | 15 | 1 | 0 | 0 | 1 | 14 |
| Subclone D | 10·0 | T | + | 100 | 1 | 2 | 0 | 3 | 4 | 0 | 6 | 8 | 15 | *39* | 9 | 3 | 0 | 0 | 0 | 10 |
| Subclone E | 5·0 | T | + | 100 | 0 | 1 | 0 | 2 | 2 | 2 | 0 | 8 | 9 | *59* | 12 | 1 | 1 | 0 | 0 | 3 |
| Subclone F | 5·0 | T | + | 100 | 1 | 0 | 0 | 2 | 2 | 0 | 3 | 6 | 5 | *37* | 32 | 6 | 5 | 0 | 0 | 1 |

* Details of cells and properties are in Kirkland, 1976, Table I.
† N = normal, T = transformed morphology at time of agar growth test and chromosome analysis.
** Modal chromosome number in italics.

FIG. 2.—Giemsa-banded karyotypes of a normal diploid cell (Chinese hamster lung is shown) and of a transformed cell derived from CHMK/H, showing a marker chromosome M. All 6 transformed kidney subclones contained this marker. ×1300.

(F and G) subclones derived from treated CHMP/E cultures (Kirkland, 1976; Table IV) are also shown in Table III. The only noticeable difference between the distributions of normal (A and E) and transformed (F and G) subclones is the presence of clear modes of 23 and 24 for A and E respectively (the subpopulation

TABLE III.—*Chromosome Distributions for Chinese Hamster Prostate Cells* (*CHMP/E*) *at the Time of Treatment with* N-*methyl*-N-*nitrosourea* (*MNU*), *and for* 4 *Subclones Isolated from Treated Plates*

| Cell culture* | Treatment (μg/ml MNU) | Morphology† | Growth in soft agar* | No. of metaphases counted | Percentage of cells containing chromosome numbers** | | | | | | | | | | | | | | | | | | |
|---|---|---|---|---|---|---|---|---|---|---|---|---|---|---|---|---|---|---|---|---|---|---|---|
| | | | | | 21 | 22 | 23 | 24 | 25 | 26 | 27 | 28 | 30–40 | 42 | 43 | 44 | 45 | 46 | 47 | 48 | 49 | 50 | >50 |
| CHMP/E | None | N | – | 50 | 0 | 4 | 6 | *66* | 2 | 0 | 0 | 0 | 0 | 0 | 0 | 0 | 0 | 0 | 0 | 22 | 0 | 0 | 0 |
| Subclone A | 50 | N | – | 50 | 2 | 14 | *52* | 8 | 0 | 0 | 2 | 0 | 0 | 0 | 0 | 6 | 0 | 6 | 0 | 6 | 0 | 0 | 4 |
| Subclone E | 100 | N | – | 50 | 0 | 2 | 6 | *54* | 6 | 0 | 0 | 0 | 0 | 0 | 0 | 2 | 0 | 4 | 0 | 26 | 0 | 0 | 0 |
| Subclone F | 100 | T | + | 50 | 2 | 2 | 22 | *28* | 6 | 6 | 2 | 0 | 4 | 0 | 0 | 2 | 0 | 10 | 2 | 6 | 2 | 0 | 6 |
| Subclone G | 100 | T | + | 50 | 2 | 2 | 4 | 2 | 6 | 10 | 2 | 2 | 18 | 4 | *14* | 8 | 8 | 2 | 4 | 2 | 2 | 2 | 10 |

* Details of cells and properties are in Kirkland, 1976, Table IV.
† N = normal, T = transformed morphology at time of agar growth test and chromosome analysis.
** Modal chromosome number in italics.

FIG. 3.—Giemsa-banded karyotype of parental Chinese hamster prostate cells (CHMP/E) containing 24 chromosomes. Reference to the normal diploid karyotype in Fig. 2 reveals the two additional chromosomes as a number 9 and a chromosome derived from two long arms of number 4. ×1100.

with 48 chromosomes in E consists mainly of endo-reduplicated cells like the parent CHMP/E), whereas there is no clear mode and a much wider spread of chromosome number in F and G.

A number of abnormalities were seen in each subclone and some are illustrated in Fig. 4 (loss of isochromosome 4, loss of Y, translocation to 7 and new chromosome in E, additional Y and trisomy 10 in F,

FIG. 4.—Giemsa-banded karyotypes of 4 subclones (2 normal and 2 transformed) isolated from CHMP/E cultures treated with *N*-methyl-*N*-nitrosourea. A marker chromosome derived from the number 4 is present only in transformed subclones F and G. $\times$ 1200.

and trisomy 5 and loss of 9 in G) but the only consistent change was a small telocentric chromosome, not present in either parental CHMP/E cells or normal subclones A and E, but found in >97% of all the metaphases analysed in detail (>70 in number) for each of the transformed subclones F and G. Giemsa banding again revealed this to be a consistent marker chromosome (Fig. 4), and again to be a fragment of the number 4 chromosome, but this time consisting of the centromere and most of the short (p) arm.

### DISCUSSION

Jackson *et al.* (1970) reported that for cultured rat cells, after a period of initial stability, the ploidy of the cells changed and abnormal chromosomes appeared, but that this was in no way related to transformation. Olinici and DiPaolo (1974) observed a number of chromosomal aberrations occurring in Syrian hamster cells treated with 7,12-dimethylbenz($\alpha$) anthracene, but saw similar changes in control cells.

The evidence presented here (Table I) indicates again that in certain culture conditions (*i.e.* single-serum medium as normally used for routine cell culture) spontaneous changes in chromosome number can occur very readily. In such a situation, we feel that any specific chromosome changes associated with a putative malignant change (induced or spontaneous) would be likely to be masked as a result of the instability of the karyotype.

We have therefore attempted to stabilize the karyotype of the Chinese hamster cells used in the present study by following the suggestions of Yerganian and Lavappa (1971), and culturing the cells in a mixed-serum medium. A comparison of the chromosome distributions in Table I shows that a mixed-serum medium has apparently caused a reduction in the proportion of aneuploidy in the Chinese hamster cell population: the system is still not perfect in that 100% diploidy is not achieved.

Using Yerganian's medium (YM) for the culture of kidney cells, we have not only been able to detect a very simple numerical change (22 to 23) associated with transformed properties (Table II) but have demonstrated that the abnormal telocentric was a consistent marker in $\geqslant$98% of all the cells in each of the 6 transformed subclones, and by Giemsa banding revealed its origin as the centromere and q arm of the number 4 chromosome (Figs. 1, 2).

Transformed subclone A derived from CHMK/H (Kirkland, 1976; Table I) was isolated from an untreated plate, and yet it shows the same karyotypic change as the other subclones isolated from 3-methylcholanthrene-treated plates. Subclone A must be a spontaneous transformant, so it could be argued that transformation following chemical treatment is merely an enhancement of a spontaneous event. This seems very likely in view of the small number (1%) of cells in the parental CHMK/H clone which contained the marker, this particular karyological change apparently being at a selective advantage over other types in the same population. All attempts to isolate normal subclones from experimental cultures failed when the cells died. This may indicate that normal diploid CHMK/H had reached the end of a natural lifetime in culture, as Hayflick (1965) observed in human cells, and at a crisis point had either died or spontaneously transformed. If this were so, it could be argued that all 6 CHMK/H subclones would effectively be spontaneously transformed, methylcholanthrene having little or no effect, and the marker chromosome would be associated with spontaneous transformation.

Transformed prostate cells (CHMP/E) showed no consistent deviation in chromosome number either from normal subclones derived from treated cultures or from the parent clone (Table III). However, an abnormal telocentric chromosome was again detected only in the 2 transformed subclones F and G, and in $>$97% of their cells. Although this was a different fragment (being the centromere and most of the p arm), it was again a consistent marker derived from the number 4 chromosome (Figs. 3, 4). No spontaneously transformed prostate cells were detected (Kirkland, 1976; Fig. 3), and therefore it is fair to assume that transformation in this case resulted from the action of *N*-methyl-*N*-nitrosourea.

Benedict *et al.* (1975) have shown that if the numerical balance between two groups (5 and 7) of the chromosomes of cultured Syrian hamster cells is disturbed in a particular direction, highly malignant cells result. We have shown here that an even more specific change involving the number 4 chromosome of the Chinese hamster occurs only in those subclones showing morphological evidence of transformation and the ability to grow in soft agar, two characteristics thought to be associated with malignant transformation. We feel that the use of a culture medium designed to stabilize the karyotype of the cultured cells is at least partly responsible for this observation, and may be a useful means of isolating specific chromosomal changes from the wealth of random changes we know to occur *in vitro* and *in vivo*. Such studies of early changes could shed a welcome light on tumour aetiology. Certainly in the Chinese hamster, chromosome 4 seems particularly susceptible to change, either spontaneously or as a result of chemical treatment *in vitro*. Our data suggest that such changes may be associated with an alteration in the properties of cells to those which some authors believe are characteristic of malignancy.

This work was supported by an A. K. Fellowship granted by the Institute of Cancer Research to D. J. Kirkland, and by grants to this Institute from the Medical Research Council and the Cancer Research Campaign.

### REFERENCES

Ahlström, U. (1974) Chromosomes of Primary Carcinomas Induced by 7,12-dimethylbenz(α) anthracene in the Rat. *Hereditas*, **78**, 235.

Benedict, W. F., Rucker, N., Mark, C. & Kouri, R. E. (1975) Correlation between Balance of Specific Chromosomes and Expression of Malignancy in Hamster Cells. *J. natn. Cancer Inst.*, **54**, 157.

DiPaolo, J. A., Popescu, N. C. & Nelson, R. L. (1973) Chromosomal Banding Patterns and *in vitro* Transformation of Syrian Hamster Cells. *Cancer Res.*, **33**, 3250.

Gallimore, P. H. & Richardson, C. R. (1973) An Improved Banding Technique Exemplified in the Karyotype Analysis of Two Strains of Rat. *Chromosoma*, **41**, 259.

Hayflick, L. (1965) The Limited *in Vitro* Lifetime of Human Diploid Cell Strains. *Expl Cell Res.*, **37**, 614.

Jackson, J. L., Sanford, K. K. & Dunn, T. B. (1970) Neoplastic Conversion and Chromosomal Characteristics of Rat Embryo Cells *in Vitro*. *J. natn. Cancer Inst.*, **45**, 11.

Kato, H. & Yosida, T. H. (1972) Banding Patterns of Chinese Hamster Chromosomes Revealed by New Technique. *Chromosoma*, **36**, 272.

Kirkland, D. J. (1976) Chemical Transformation of Chinese Hamster Cells. I. A Comparison of Some Properties of Transformed Cells. *Br. J. Cancer*, **34**, 134.

Kirkland, D. J. & Venitt, S. (1976) Cytotoxicity of Hair Colourant Constituents: Chromosome Damage Induced by Two Nitrophenylenediamines in Cultured Chinese Hamster Cells. *Mutation Res.*, **40**, 47.

Mitelman, F., Mark, J., Levan, G. & Levan, A. (1972*a*) Tumour Etiology and Chromosome Pattern. *Science, N.Y.*, **176**, 1340.

Mitelman, F., Mark, J. & Levan, G. (1972*b*) Chromosomes of Six Primary Sarcomas Induced in the Chinese Hamster by 7,12-dimethylbenz(α)-anthracene. *Hereditas*, **72**, 311.

Olinici, C. D. & DiPaolo, J. A. (1974) Chromosome Banding Patterns of Rat Fibrosarcomas Induced by *in Vitro* Transformation of Embryo Cells or *in Vivo* Injection of Rats by 7,12-dimethylbenz(α) anthracene. *J. natn. Cancer Inst.*, **52**, 1627.

Yerganian, G. & Lavappa, K. S. (1971) Procedures for Culturing Diploid Cells and Preparation of Meiotic Chromosomes from Dwarf Species of Hamsters. In *Chemical Mutagens, Principles and Methods for their Detection*, Vol. 2, New York and London: Plenum Press. p. 387.